# A high quality, high molecular weight DNA extraction method for PacBio HiFi genome sequencing of recalcitrant plants

**DOI:** 10.1186/s13007-023-01009-x

**Published:** 2023-04-29

**Authors:** Kanae Nishii, Michael Möller, Robert G. Foster, Laura L. Forrest, Nathan Kelso, Sadie Barber, Caroline Howard, Michelle L. Hart

**Affiliations:** 1 Royal Botanic Garden Edinburgh, 20A Inverleith Row, Edinburgh, EH3 5LR UK; 2 Kanagawa University, 2946 Tsuchiya, Hiratsuka, Kanagawa 259-1293 Japan; 3 Edinburgh Genomics, The University of Edinburgh, Charlotte Auerbach Rd., Edinburgh, EH9 3FL UK; 4 Wellcome Sanger Institute, Wellcome Trust Genome Campus, Hinxton, Saffron Walden, CB10 1RQ UK

**Keywords:** DNA extraction, Genome sequencing, Gesneriaceae, Long-read sequencing, Next-generation sequencing, PacBio HiFi, SMRTbell™ library, *Streptocarpus*

## Abstract

**Background:**

PacBio HiFi sequencing provides highly accurate long-read sequencing datasets which are of great advantage for whole genome sequencing projects. One limitation of the method is the requirement for high quality, high molecular weight input DNA. This can be particularly challenging for plants that frequently contain common and species-specific secondary metabolites, which often interfere with downstream processes. Cape Primroses (genus *Streptocarpus*), are some of these recalcitrant plants and are selected here as material to develop a high quality, high molecular weight DNA extraction protocol for long read genome sequencing.

**Results:**

We developed a DNA extraction method for PacBio HiFi sequencing for *Streptocarpus grandis* and *Streptocarpus kentaniensis.* A CTAB lysis buffer was employed to avoid guanidine, and the traditional chloroform and phenol purification steps were replaced with pre-lysis sample washes. Best cells/nucleus lysis was achieved with 4 h at 58 °C. The obtained high quality and high molecular weight DNAs were tested in PacBio SMRTBell™ library preparations, which resulted in circular consensus sequencing (CCS) reads from 17 to 27 Gb per cell, and a read length N50 from 14 to 17 kbp. To evaluate the quality of the reads for whole genome sequencing, they were assembled with HiFiasm into draft genomes, with N50 = 49 Mb and 23 Mb, and L50 = 10 and 11. The longest contigs were 95 Mb and 57 Mb respectively, showing good contiguity as these are longer than the theoretical chromosome length (genome size/chromosome number) of 78 Mb and 55 Mb, for *S. grandis* and *S. kentaniensis* respectively.

**Conclusions:**

DNA extraction is a critical step towards obtaining a complete genome assembly. Our DNA extraction method here provided the required high quality, high molecular weight DNA for successful standard-input PacBio HiFi library preparation. The contigs from those reads showed a high contiguity, providing a good starting draft assembly towards obtaining a complete genome. The results obtained here were highly promising, and demonstrated that the DNA extraction method developed here is compatible with PacBio HiFi sequencing and suitable for de novo whole genome sequencing projects of plants.

**Supplementary Information:**

The online version contains supplementary material available at 10.1186/s13007-023-01009-x.

## Background

PacBio HiFi long-read DNA sequencing, so-called circular consensus sequencing (CCS), greatly improves the accuracy of third generation long-read sequencing compared to original continuous long-read (CLR) sequencing [1]. This method requires high molecular weight, high quality DNA as input material for successful standard library preparation, with an average required DNA input size of > 40–50 kb [2, 3].

DNA extraction from plant tissues frequently poses challenges due to the presence of secondary metabolites such as polysaccharides and phenolic compounds (reviewed in [4]). For this reason, many optimised DNA extraction methods have been developed for NGS downstream applications (e.g. more than 30 references in *Plant Methods* in September 2022, e.g., [5, 6]), although only one of these includes HiFi, in the study of the model plant *Fragaria* (accessed September 2022; [7] cited in [8]).

Cape Primroses (*Streptocarpus*) are non-model plants studied for their highly variable vegetative form [9, 10], which is due to shifting meristem activities [11–13]. The first *Streptocarpus* genome, of *S. rexii*, a species with a 1C genome size of *ca.* 929 Mb and 2*n* = 32 chromosomes [14], was recently published [15] using Oxford Nanopore Technologies (ONT) PromethION sequencing with a DNA extraction method specifically developed for the ONT genome sequencing of *Streptocarpus* [15, 16]. However, the DNA extraction protocol for PacBio HiFi sequencing has specific requirements; for example, the guanidine lysis buffer, phenol and chloroform purification need to be avoided as they interfere with sequencing performance [3]. Therefore, previous DNA extraction methods suitable for ONT sequencing are not suitable for PacBio HiFi sequencing [16]. In addition, explorative attempts at DNA extraction from *Streptocarpus* with the former method resulted in fragment size peaks below 40 kb (Additional file 1: Figure S1, Additional file 2: Table S1) and thus did not satisfy the PacBio HiFi requirement of > 40–50 kb [2, 3]. We hypothesized that this fragmentation was due to the strong lysis condition of the ONT protocol, and thus explored alternatives here. We developed a DNA extraction protocol specific for PacBio HiFi sequencing, using *Streptocarpus grandis* (1C ≈ 1261 Mb, 2*n* = 32 [14]) and *Streptocarpus kentaniensis* (1C ≈ 876 Mb, 2*n* = 32 [14]) as the test material and performed PacBio HiFi sequencing to evaluate the suitability of the extracted DNA for PacBio SMRTbell™ library preparation, HiFi sequencing and genome assembly.

## Results and discussion

### Developing DNA extraction method for PacBio HiFi sequencing

The protocol described here was developed from a previous method used for ONT sequencing [15, 16]. However, this protocol uses a guanidine-based lysis buffer (Qiagen buffer G2, 800 mM guanidine hydrochloride; 30 mM Tris-HCl, pH 8.0; 30 mM EDTA, pH 8.0; 5% Tween 20; 0.5% Triton X-100), unsuitable for PacBio HiFi sequencing [3], and was replaced with a CTAB buffer (see “Methods” section) [17, 18]. The chloroform and phenol purification traditionally combined with CTAB [17, 18] was avoided as it also interferes with PacBio HiFi sequencing [3]. We tested two lysis time regimes, 50 °C overnight [16], and 58 °C for 4 h. As a rather minor adjustment, only the 100 µm pore size nylon mesh was used instead of both of 100 µm and 40 µm pore size nylon meshes consecutively as used in a previous protocol [16], since this change substantially increased the final DNA quantity extracted (K. Nishii personal observation).

**Fig. 1:**
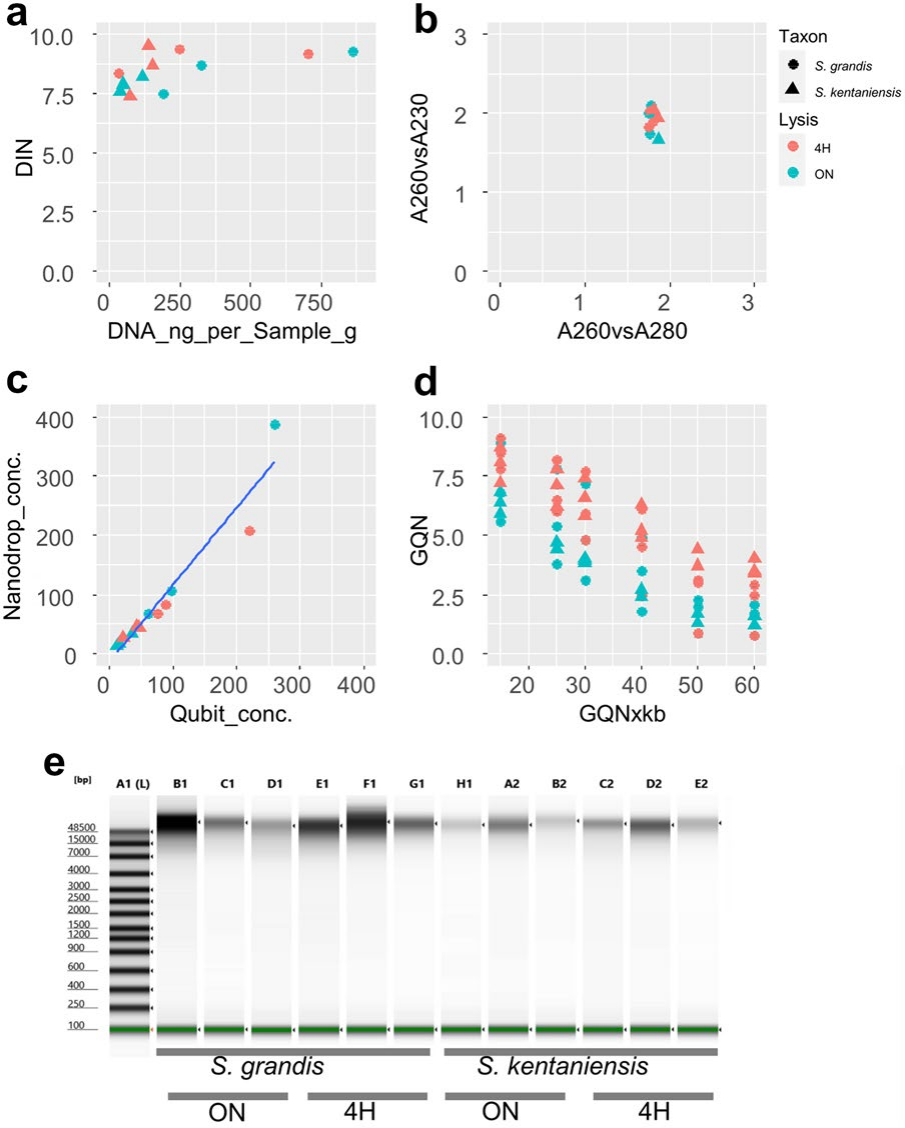
Quality of small scale DNA extractions using the protocol developed in this study. DNA was extracted in triplicates from two species of *Streptocarpus*, *S. grandis* and *S. kentaniensis*, using two different lysis conditions: 4H—4 h at 58 °C, ON—overnight at 50 °C. **a** Scatter plot of TapeStation Genomic DIN values (y-axis) versus obtained DNA in ng per g leaf sample (x-axis). **b** Scatter plot of spectrophotometer ratios, A260/A230 (A260vsA230) versus A260/A280 (A260vsA280). **c** Scatter plot of DNA concentration (ng/µl) quantified with fluorometer (Qubit_conc) versus spectrophotometer (Nanodrop_conc). **d** Scatter plot of Femto Pulse GQN analyses. GQN values (y-axis) indicate the proportion of the fragment quantity of the total DNA quantity of the GQNxkb threshold length (x-axis). For example, 50 on the x-axis indicates a fragment size of GQN_50kb_ and the proportion of DNA quantity > 50 kb is shown on the y-axis. **e** TapeStation gel image of extracted DNA. Lanes: A1 ladder, B1-G1 *S. grandis*, H1-E2 *S. kentaniensis,* B1-D1 and H1-B2 ON lysis, E1-G1 and C2-E2 4H lysis

**Fig. 2:**
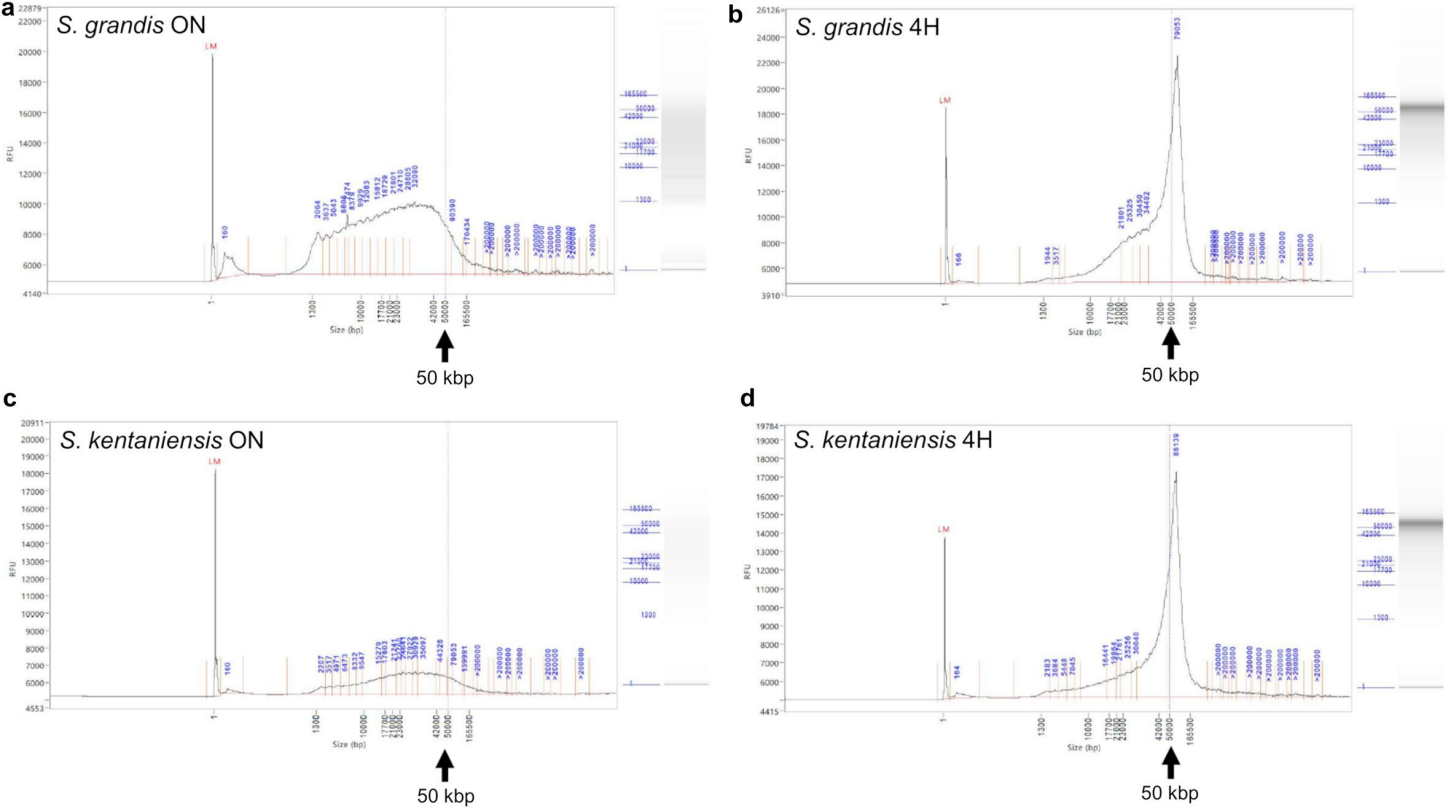
Representative results of Femto Pulse runs on *Streptocarpus* small scale DNA extracted in this study. **a**, **b** *S. grandis* **c**, **d** *S. kentaniensis* **a**, **c** Lysis condition overnight at 50 °C **b**, **d** Lysis condition for 4 h at 58 °C

To test the robustness of the method, we performed triplicate replicates on *S. grandis* and *S. kentaniensis* with two lysis conditions mentioned above. The TapeStation (Agilent Technologies) DIN values for the extracted DNA for both species and lysis regimes were > 7.4 and the highest, 9.3 (Fig. 1a). The DNA for most samples showed ideal spectrophotometer values, A260/A280 (> 1.8) and A260/A230 (> 2.0) (Fig. 1b), with only a few samples slightly lower values of around A260/A280 ~ 1.7 and A260/A230 ~ 1.8, indicating the presence of a small amount of contaminants. The fluorimeter DNA quantification and the spectrophotometer DNA quantification were in very high agreement (Fig. 1c), indicating low amounts of contaminants such as RNA and also polysaccharides [3]. The DNA fragment size distribution evaluated with Femto Pulse [19] indicated that lysis for 4 h at 58 °C resulted in longer fragments (Figs. 1d, 2), indicating this to be better for PacBio HiFi sequencing purposes than overnight lysis, and thus this was used in the protocol below.

**Fig. 3:**
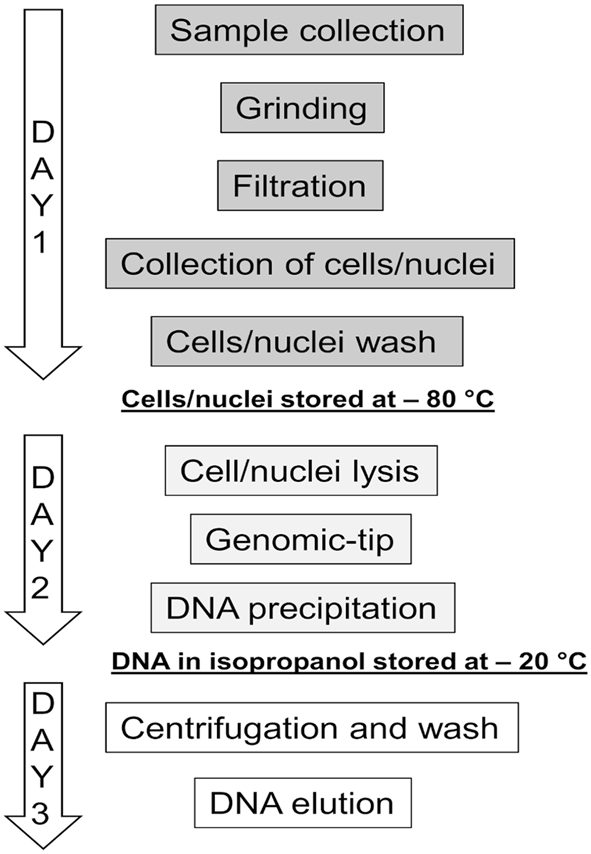
Schematic overview illustration of the DNA extraction method for PacBio HiFi sequencing developed in this study

**Fig. 4:**
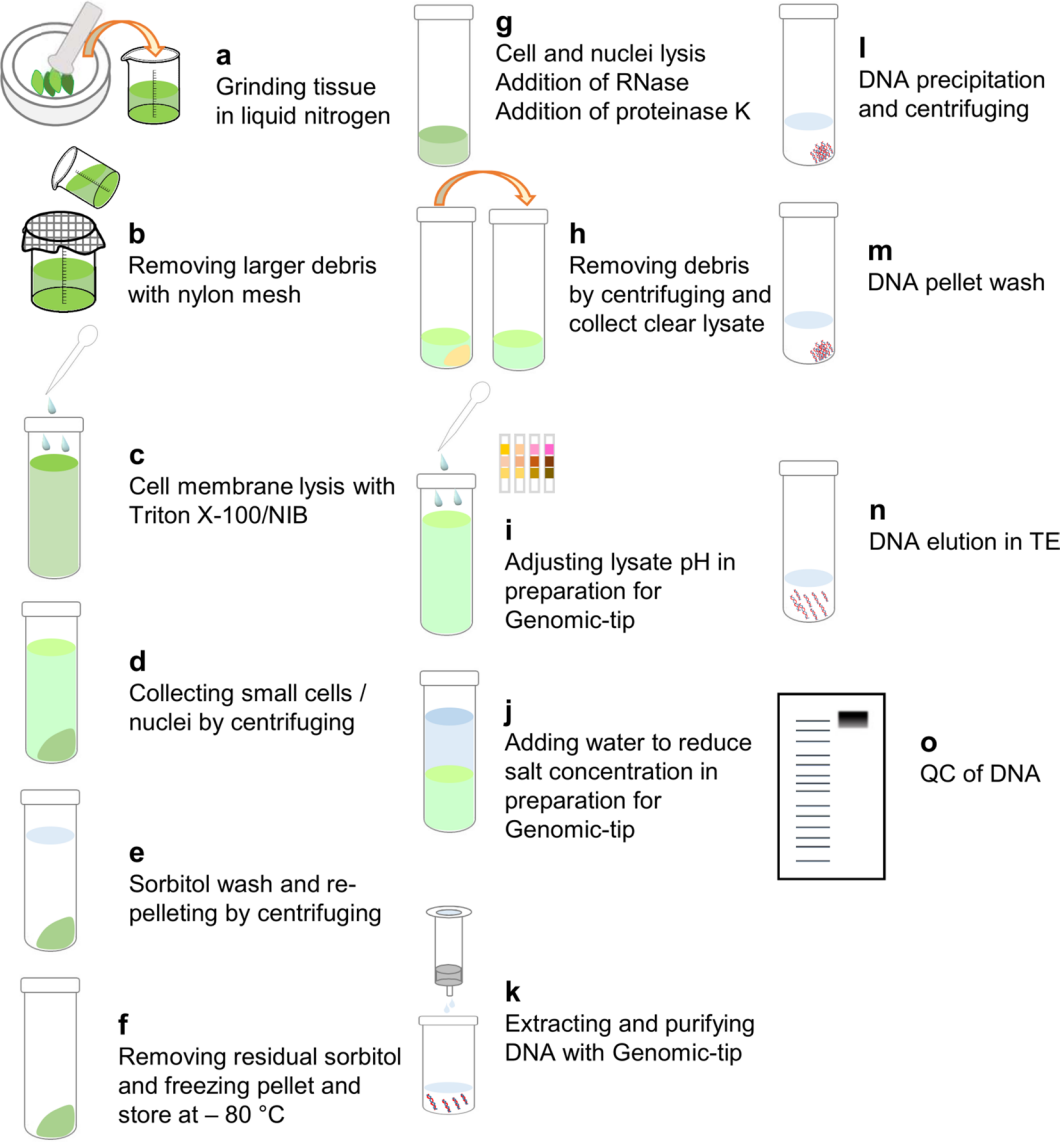
Schematic illustration of the steps involved in the PacBio HiFi DNA extraction protocol. Steps a–o explained in text under ‘Procedure’

Among the triplicate DNA extractions here, the nanograms of DNA yielded per gram starting leaf sample tissue (DNA-ng/Sample-g) varied between species, lysis condition and replicates (Fig. 1a, Additional file 2: Table S2). For *S. grandis* in particular, it varied between 29.33–860.89 DNA-ng/Sample-g. This might have been due to the great variation in cell size across the leaf of this unifoliate plant, being small in the proximal meristematic area and large in the distal differentiated area of the lamina [13], and two samples (KN327, KN331; Additional file 2: Table S2) may have contained predominantly meristematic tissue of the immature plants used here. The values for *S. kentaniensis* were more uniform, likely due to the more uniform cell sizes of the fully expanded leaves used for this rosulate species. The workflow of the protocol is provided in Fig. 3, and the full protocol is described in detail in the “Methods” section below and illustrated in Fig. 4.

### Testing the extracted DNA for PacBio HiFi library preparation and sequencing

For the PacBio HiFi sequencing run, we extracted larger amounts of DNA using the method established here (Figs. 1, 2, 3 and 4) from *S. grandis* and *S. kentaniensis*, and obtained 21.3 µg and 22.3 µg of DNA from 48 g and 45 g starting material respectively. This was sufficient for multiple library preparations and quality controls (Additional file 2: Table S3). The PacBio HiFi standard protocol requires 5 µg DNA per library in the standard protocol [20]. This is excluding DNA QC steps and AMPpure XP/PB bead cleaning and normalization steps, and thus usually more than 5 µg raw DNA is required for the whole procedure [20]. In this study, the extracted DNA was processed for sequencing at Edinburgh Genomics. 7.5 µg raw DNA per library was purified and resulted in 5 µg normalized DNA ready for subsequent library preparation (see details in the “Methods” section), and sequencing (Additional file 1: Figure S3). The library preparation provided sufficient DNA for two SMRT™ cells per species and sequencing achieved > 30× coverage for the 1C genome sizes of *ca.* 1260 Mb (*S. grandis*) and *ca.* 876 Mb (*S. kentaniensis* [14]) respectively. From 17 to 27 Gb CCS read data was obtained per SMRT™ cell (Table 1, Additional file 2: Table S4), comparable with previous plant PacBio HiFi studies (Table 2). The longest read was 57,931 bp and the read N50 = 17,900 bp in *S. grandis*, and the longest read, 46,286 bp and N50 = 14,227 bp in *S. kentaniensis* respectively (Table 1, Additional file 2: Table S4). To examine the read performance in the genome assembly, draft genome assemblies were constructed with HiFiasm [21]. For *S. grandis*, the L50 was 10 and L75 = 18, and for *S. kentaniensis*, L50 = 11 and L75 = 22 (Table 3). This is considerably high, considering the haploid (*n*) and basic (*x*) chromosome number for both species is *n* = x = 16. In these draft assemblies, the longest contig in *S. grandis* was > 94 Mbp and in *S. kentaniensis* > 56 Mbp (Table 3, [14]), which are longer than the theoretical chromosome lengths of *ca*. 78 Mbp (*S. grandis*, 1C ≈ 1261 Mb, *n* = 16) and *ca.* 54 Mbp (*S. kentaniensis*, 1C ≈ 876 Mb, *n* = 16). Thus, this demonstrated that the DNA extraction protocol developed in this study was suitable for whole genome sequencing projects in *Streptocarpus*, as it leads to high quality HiFi reads and high quality draft genome assemblies.

**Table 1:** Statistics of the PacBio HiFi long-read sequencing results

	*S. grandis*	*S. kentaniensis*
Mean read length (bp)	17,751.6	15,551.3
Mean read quality	32.8	33.3
Median read length (bp)	16,944.0	14,970.0
Median read quality	32.8	33.1
Number of reads	1,308,850.5	1,478,419.5
Read length N50 (bp)	17,900.0	14,227.0
Longest read length (bp)	54,989.5	45,411.5
STDEV read length	4096.7	3355.1
Total bases per cell (bp)	23,234,405,706.0	22,573,446,894.5

Sequencing results of the PacBio HiFi long-read sequencing of *Streptocarpus grandis* and *S. kentaniensis* assessed with NanoPlot. Values are the average of two SMRT™ cells per species

**Table 2:** Comparison between plant PacBio HiFi long-read sequencing results

Taxon	DNA extraction method	Amount per cell (Gb)	Mean read length (kb)	References (DNA extraction)
*Zea mays*	CTAB	24.1	15.6	Hon et al. 2020 [8] (Doyle and Doyle 1987 [18])
*Fragaria* × *ananassa*	NI, Modified CTAB	29.7	21.7	Hon et al. 2020 [8] (Li et al. 2020 [7])
*Solanum lycopersicum*	MN NucleoBond HMW DNA kit	20.3	18.3	van Rengs et al. 2022 [22]
*Streptocarpus grandis*	NI, CTAB, Qiagen Genomic-Tip	23.2	17.9	This study
*Streptocarpus kentaniensis*	NI, CTAB, Qiagen Genomic-Tip	22.6	15.6	This study

Comparison between the current data and published plant PacBio HiFi datasets

*NI* Nuclei isolation or similar filtration, *MN* Macherey–Nagel

**Table 3:** Statistics of draft *Streptocarpus* genome assemblies

Assembler	*S. grandis*	*S. kentaniensis*
Num. of contigs (> = 0 bp)	5653	1168
Num. of contigs (> = 25,000 bp)	5482	1049
Num. of contigs (> = 50,000 bp)	1661	194
Total length (> = 0 bp)	1,253,163,518	710,000,308
Total length (> = 25,000 bp)	1,249,324,457	707,346,651
Total length (> = 50,000 bp)	1,105,422,048	677,448,832
Total length/genome size (%)	99.5	81.1
Estimated 1C genome size (Mb) [13]	1261	876
Haploid chromosome number (*n*) [13]	16	16
Largest contig length (bp)	94,998,679	56,620,953
Theoretical chromosome size (Mb)	78.8	54.8
GC (%)	38.7	38.4
N50	48,948,889	23,428,243
N75	23,325,262	9,463,912
L50	10	11
L75	18	21

Draft genome assembly quality control (QC) results to evaluate the DNA extraction method for whole genome sequencing projects. Reads were assembled with HiFiasm and examined with Quast

### The pros and cons of the HiFi DNA extraction protocol

This protocol has been proven to generate high quality, high molecular weight DNA suitable for PacBio HiFi long-read sequencing. However, it requires relatively large amounts of fresh plant material: to obtain ~ 5 µg normalized and purified input DNA for one PacBio HiFi library preparation it requires more than 10 g of *Streptocarpus* leaf tissue. For study material that exists as living plants in botanical gardens or is accessible nearby in abundance in nature, obtaining 10 g leaf tissue is feasible for most larger herbaceous plants, shrubs, and trees. In the case of *S. grandis*, a unifoliate with a large leaf of up to 38 × 33 cm [23–25], one plant was sufficient to provide enough material for the large scale DNA extraction. On the other hand, this will be far more challenging for small herbs and bryophytes. For animals, the establishment of cell culture from endangered species can successfully provide sufficient material for DNA extraction [26]. For plants, clonal propagation or the establishment of callus or cell lines could generate more material from small starting amounts of tissue (e.g., *Sphagnum* [27]).

Although this extraction protocol relies on larger amounts of leaf material, it is very cost effective. Grinding leaf tissues in liquid nitrogen and filtering in nuclei isolation buffer are relatively inexpensive steps, and the latter substantially reduces the amount of material loaded onto Genomic-tip columns and contributes significantly to the increase in DNA quality here.

The procedure outlined here also allows breaks in the DNA extraction protocol, such as storage of the nuclei/small cell filtrate for at least several days at − 80 °C after snap freezing in liquid nitrogen, to ease work schedules. This also allows the filtrates from several filtrations to be collected first before processing them together for larger scale DNA extraction, for larger genomes or where sufficient material becomes available over time or the yield/g tissue is low.

There are possible improvements to the protocol. The liquid nitrogen grinding step with mortar and pestle is labour intensive, particularly for large, high sample throughput projects such as the Darwin Tree of Life (https://www.darwintreeoflife.org/). Pestle and mortar can be replaced with a mixer mill (QIAextractor, Qiagen, Hilden, Germany) and liquid nitrogen-frozen leaf material in Eppendorf tubes, although this still requires the handling of many tubes. The Cryomill (Retsch, Haan, Germany) with 50 ml grinding jars might be a good option to replace the most labour-intensive hand grinding step. On the other hand, for the sequencing of a few genomes, the method described here is workable since the DNA extraction of one sample takes just 3 days. For samples where tissue input is very limited, both low and ultra-low DNA input PacBio Hifi protocols are now available [28, 29], only requiring ~ 300 ng or ~ 10 ng DNA, respectively, and hence require a smaller amount of starting leaf tissues, although these options might result in reduced sequencing contiguity and affect genome assembly (“Where possible, the standard HiFi workflow run on the Sequel^®^ II System gives you the highest quality results for both genome assembly and human variant detection projects” [30]). In this study, we employed a short lysis for 4 h at 58 °C for the large-scale DNA extraction. This step could still be improved. However, a shorter, ~ 2 h lysis did not generate sufficient quantities of DNA (K. Nishii, personal observation), and thus, a fine-tuning of the lysis temperature against time might improve balancing the DNA molecular weight and quantity.

With the steady progress in genome sequencing and assembly methodology over the past years, the bottleneck will be the supply of high molecular weight, high quality DNA. There is a high demand for quick, simple and reproducible DNA extraction protocols, particularly for plants, and future developments are expected to reduce the time and labour involved while maintaining or improving quality.

## Conclusions

The protocol presented here was successful in the extraction of high molecular weight, high quality DNA from recalcitrant plants that, following PacBio HiFi long-read library preparation and sequencing, allowed the construction of draft genome assemblies with near-chromosome length contigs. The presented protocol was developed for *Streptocarpus* and worked well for two species of this difficult to extract genus, but this protocol has the potential to be adapted to other plants recalcitrant to the extraction of high molecular weight, high quality DNA for long-read genome sequencing.

## Methods

### Plant material

*Streptocarpus grandis* (RBGE lineage 19771210) and *Streptocarpus kentaniensis* (RBGE lineage 19951992) were cultivated in the glasshouses at the Royal Botanic Garden Edinburgh.

### DNA extraction method for *Streptocarpus* PacBio HiFi long-read sequencing

To develop the protocol, small scale extractions were carried out with approx. 3 g leaf tissue as input material. Two species, *Streptocarpus grandis* and *S. kentaniensis* were tested in biological triplicates. Lysis conditions of (1) 50 °C overnight and (2) 58 °C for 4 h were compared. For large-scale extractions for genome assemblies, 48 g (*S. grandis*) and 45 g (*S. kentaniensis*) of leaf tissue was used as starting material respectively.

The DNA extraction protocol is described below, while a more detailed step-by-step protocol is deposited in protocols.io (DOI: 10.17504/protocols.io.x54v9dm7pg3e/v1). Buffers and reagent amounts were adapted to match the starting material. For small scale 3 g extractions, a 1/10 scale reduction in volumes was used for the pre-lysis step, i.e. instead of 10 Falcon tubes, one Falcon tube per sample was used, and Qiagen Genomic-tip 20/G columns were used following to the manufacturer’s protocol, instead of Genomic-tip 100/G columns in the large scale DNA extraction.

### Buffers and reagents

Nuclei isolation base (NIB) buffer: 10 mM Tris-HCl pH 8.0, 10 mM EDTA pH 8.0, 500 mM sucrose, 100 mM KCl, in deionized water and autoclaved.

Nuclei isolation (NI) buffer: NIB buffer supplemented with 4 mM spermidine, 1 mM spermine, and 0.1% *β*-mercaptoethanol. Prepared on the day of DNA extraction and left on ice for precooling.

Triton X-100/NIB: 10% Triton X-100 in NIB buffer. Briefly heated in a water bath at 50 °C to dissolve Triton X-100. Stored at 4 °C.

Sorbitol buffer base solution: 100 mM Tris-HCl pH 8.0, 5 mM EDTA pH 8.0, 700 mM sorbitol in deionized water and autoclaved. The original sorbitol concentration in the buffer is 350 mM [31], but has been increased to 700 mM in the present protocol.

Sorbitol buffer: Sorbitol buffer base solution supplemented with 1% PVP40 and 0.2% *β*-mercaptoethanol. To be prepared on the day of DNA extraction.

CTAB lysis buffer: 100 mM Tris-HCl pH 8.0, 20 mM EDTA pH 8.0, 1.4 M NaCl, 2% CTAB (v/v), in deionized water and autoclaved.

0.25N HCl

RNase A (100 mg/ml).

Proteinase K (Qiagen, #19157).

Polyvinyl polypyrrolidone (PVPP).

Isopropanol.

70% ethanol.

Low TE buffer (0.1×).

### Plastics and genomic-tip columns and buffers

This protocol uses ordinary pipettes (max volume: 20 µl, 200 µl, 1 ml) from Gilson (Middleton, WI, USA) or Rainin™ (Metter-Toledo Rainin, Oakland, CA, USA) and respective pipette tips for general pipetting, while for the lysate and steps involving DNA, wide-bore 1 ml pipette tips (Metter-Toledo Rainin) were used. Falcon tubes (50 ml), Eppendorf tubes (2 ml, Eppendorf, Hamburg, Germany), and Eppendorf LoBind tubes (1.5 ml) were used throughout the protocol. Corning^®^ Cell Strainers (Corning Inc. Corning NY, USA) of 100 µm pore size were used for small scale extractions. Qiagen Genomic-tip 20/G and 100/G (#10223, #10243, Qiagen, Hilden, Germany) columns were used in combination with the modified CTAB buffer described above, and buffer QBT, buffer QC, and buffer QF as purchased from Qiagen. Hydrogen-ion exponent (pH) indicating strips (Fisherbrand™ pH 4.5–10; Thermo Fisher Scientific, Waltham, MA, USA) were used to check the pH of the lysate.

### Procedure

Note: This protocol is designed for the processing of ~ 30–50 g of *Streptocarpus* leaf material. In this study, 45 g and 48 g of leaf tissue was processed with the same protocol.

0. Before starting, prepare 400 ml NI buffer in a glass bottle or beaker, and 100 ml sorbitol buffer in three 50 ml Falcon tubes, and leave them all on ice. Fix the 100 µm pore size nylon mesh with tape or a string near the top of an empty 500 ml glass beaker, or use Corning^®^ Cell Strainers (pore size 100 µm) over the 50 ml Falcon tube.
1. Prepare 400 ml NI buffer in a > 500 ml glass beaker, and leave on ice.
2. Grind fresh leaf tissue in liquid nitrogen, using a mortar and pestle. Grinding of each leaf batch was repeated three times carefully adding liquid nitrogen before the material thaws. Immediately add the ground sample to the NI buffer prepared at step 1. Grind batches of 1–2 g leaf tissue at a time, up to the total ~ 30–50 g (Fig. 4a).
3. Filter the sample-NI buffer suspension through 100 µm pore size nylon mesh prepared at step 0 in order to remove tissue debris. Carry out all procedures on ice (Fig. 4b).
4. Divide the filtrate equally between ten 50 ml Falcon tubes, and keep on ice. (*Note*: For small scale extractions (1/10), the filtrate is not divided here and one 50 ml Falcon tube per sample is used.)
5. Add 1/20 volume of 10% Triton X-100/NIB to each of the Falcon tubes prepared at step 4. Gently mix by inverting the tubes (Fig. 4c).
6. Centrifuge tubes at 2000×*g* for 10 min at 4 °C to collect small cells/nuclei (Fig. 4d).
7. Discard supernatant gently by decantation, without losing pellet.
8. Add 10 ml sorbitol buffer to each tube and gently mix to dissolve pellet (Fig. 4e).
9. Centrifuge tubes at 3000×*g* for 10 min at 4 °C to re-pellet the small cells/nuclei (Fig. 4e).
10. Discard supernatant by decantation. (Optionally, repeat sorbitol buffer wash, steps 8–10, until supernatant is clear.)
11. To remove sorbitol buffer completely, invert tubes briefly on dry paper tissue, taking care not to lose the pellet. The pellets in the tubes can now be flash frozen in liquid nitrogen and stored at − 80 °C for a few days (Fig. 4f).
12. Add 3 ml CTAB lysis buffer, 1% PVPP (v/v), and 12 µl RNase A to each tube, and mix gently by stirring with a pipette tip to dissolve the pellet. Incubate at 58 °C for 20 min for cell/nuclei lysis (Fig. 4g).
13. Add 60 µl proteinase K to each tube. Incubate 4 h (3–5 h) at 58 °C. Occasionally remove and gently shake tubes (Fig. 4g).
14. Centrifuge at 4400×*g* for 10 min. Carefully collect the clear lysate by pipetting into a new 50 ml Falcon tube, avoiding any debris (Fig. 2h).
15. Move the remaining debris/lysate from the Falcon tubes to 2 ml Eppendorf tubes, and centrifuge at 11,000 rpm for 5 min. Pipette off this clear lysate to the same new Falcon tube as at step 14. In total, approx. 30 ml lysate should be obtained.
16. Adjust lysate with 0.25 N HCl to pH 7.0–7.5 in preparation for Genomic-tip 100/G. Check pH using pH indicating strips. Add 1 ml or less 0.25 N HCl at a time, and check each time with pH indicating strips (Fig. 4i).
17. Divide lysate equally into two new 50 ml Falcon tubes. Add an equal volume of nuclease-free water in preparation for Genomic-tip 100/G (Fig. 4j). (*Note*: Do not centrifuge after adding water. Low salt concentrations tend to form CTAB-DNA solid complexes [32] and these will be removed during centrifugation.)
18. Proceed with the Qiagen Genomic-tip 100/G following the manufacturer’s instructions (Fig. 4k). Set up six empty 50 ml Falcon tubes, labelled “QBT”, “Sample”, “QC1”, “QC2”, “QC3”, “Final DNA”. Set up three sets of Genomic-tip 100/G and tubes for the ~ 60 ml lysate. Use labelled tubes with corresponding buffers. Equilibrate columns with buffer QBT, load one third of the lysate obtained at step 17 (*ca.* 20 ml) to each Genomic-tip 100/G. For the buffer QC wash, repeat three times, instead of the Qiagen protocol recommendation of two. For the final DNA elution, load 5 ml QF buffer, pre-warmed to 50 °C, to each column. (*Note*: For small scale extractions, one Genomic-tip 20/G was used here and loaded 1 ml QF buffer.)
19. Divide the eluted DNA by adding 1 ml into each 2 ml tube. Add 0.7 volumes (0.7 ml) ice-cold isopropanol for DNA precipitation. Gently invert, mix, and leave at − 20 °C overnight (Fig. 4l).
20. Centrifuge at 11,000 rpm for 10 min to pellet the DNA (Fig. 4l).
21. Discard the supernatant and add 1 ml 70% ethanol to wash the DNA (Fig. 4m).
22. Centrifuge at 11,000 rpm for 10 min to re-pellet the DNA (Fig. 4m).
23. Discard the supernatant and air dry the pellet by inverting the tubes on clean paper tissue. Heat the tubes to 37 °C for 10 min to speed up evaporation of residual ethanol, but never over-dry.
24. Add 15 µl Low TE buffer to each tube (Fig. 4n). (*Note*: For small scale extractions, 10 µl Low TE was added to the tube).
25. Incubate the tubes at 50 °C, with agitation at 300 rpm for 1 h, to dissolve the DNA. Collect the DNA into one 1.5 ml LoBind Eppendorf tube (Fig. 4n).
26. Perform appropriate QC steps for the extracted high molecular weight DNA (Fig. 4o).

### DNA quality control (QC)

The quantity and concentration of DNA present was measured in triplicate using a Qubit dsDNA BR Assay Kit (Thermo Fisher Scientific, Waltham, MA, USA, Q32853) and a Qubit 3.0 Fluorometer (Thermo Fisher Scientific, Q33216). Absorbance values (to measure contamination) were taken using a NanoDrop ND-1000 Spectrophotometer (Thermo Fisher Scientific). Size measurements were taken with either a Tapestation 2200 (Agilent Technologies, Santa Clara, CA, USA) and Genomic DNA Screentape (Agilent Technologies, 5067-5365) and Genomic DNA Reagents (Agilent Technologies, 5067-5366), or a Femto Pulse (Agilent Technologies) and the Genomic DNA 165 kb Kit (Agilent Technologies, FP-1002-0275). To size-evaluate the high molecular weight DNA, GQN (Genomic quality number) analyses of Femto Pulse runs were carried out using Prosize (Agilent Technologies) in gDNA mode; for example, a GQN_50kb_ of 0.5 indicates that 50% of fragments are longer than 50 kb in the total DNA [19].

### PacBio HiFi sequencing and quality control

The extracted DNA was processed at Edinburgh Genomics (University of Edinburgh, Edinburgh, UK). DNA was initially quality controlled, as detailed above, to check the size, concentration and amount of DNA, concentration and amount of RNA and absorbance values to measure contamination. Once the DNA had passed these checks, it entered library preparation using a modified version of the PacBio published HiFi library preparation protocols.

15 µg of the input DNA was purified using AMPure XP/PB beads (PacBio, Menlo Park, CA, USA, 100-265-900) at a 1× volume ratio, eluting into 100 µl of EB (PacBio, 101-633-500). The concentration of this elution was measured using a Qubit dsDNA BR Assay Kit and a 83 ng/µl dilution was prepared. This dilution was sheared using a Megaruptor^®^ 3 (Diagenode) and Megaruptor^®^ 3 Shearing Kit (Diagenode, E07010003) in two rounds, using speed setting 29 and then speed setting 30. The concentration of the sheared sample was measured using a Qubit dsDNA HS Assay Kit (Thermo Fisher Scientific, Q32854) and 10 µg was made up to 100 µl with EB (PacBio). This was split into two 5 µg, 50 µl aliquots which were taken through the PacBio Protocol ‘Preparing HiFi SMRTbell^®^ Libraries using the SMRTbell Express Template Prep Kit 2.0’ (PacBio, PN 101-853-100 Version 05 August 2021) until ‘Adapter Ligation’ using the SMRTbell Express Template Prep Kit 2.0 (PacBio, 100-938-900). At this point, the samples were transferred to an older version of the same protocol (PN 101-853-100 Version 03 January 2020) which uses a different Nuclease Mix (SMRTbell^®^ Enzyme Clean up Kit, PacBio, 101-746-400) and has been found to be less harsh on the libraries (R. Foster, unpublished results).

Following this, a 1× AMPure XP/PB bead clean-up was performed and the purified libraries were pooled and size-selected using a BluePippin (Sage Science), on a single lane of a 0.75% gel (Sage Science, BLF7510) with Marker S1 (Sage Science) and the 6–10 kb vs3 programme using a 10–35 kb range. The elution from the BluePippin was concentrated using 1× AMPure XP/PB beads, eluting in 15 ul EB (PacBio). 1 µl of this elution was used to measure the concentration, using a Qubit dsDNA HS Assay Kit (Thermo Fisher Scientific, Q32854). A further 1 µl was used to prepare a 0.4 ng/µl dilution, which was used for sizing the library on a Femto Pulse (Agilent Technologies), using the Genomic DNA 165 kb Kit (Agilent Technologies, FP-1002-0275).

Libraries were prepared for loading using the concentration and average size measured above, following the instructions generated by the SMRT Link software (PacBio, version 10.1.0.119588) and the ‘HiFi Reads’ application. The Sequel^®^ II Binding Kit 2.2 (PacBio, 101-894-200) was used to prepare the library for loading, alongside the Sequel^®^ II DNA Internal Control Complex 1.0 (PacBio, 101-717600). Once prepared for loading, the library was loaded and sequenced using a Sequel^®^ II Sequencing Kit 2.0 (PacBio, 101-820-200) and 2× SMRT™ Cell 8 M (PacBio, 101-389-001) on the Sequel^®^ II Sequencer (PacBio).

Read QC was carried out using NanoPlot [33] and draft assemblies were constructed using HiFiasm [21], with default settings. The results were evaluated using Quast [34].

## Supplementary Information

**Additional file 1: Figure S1.** DNA extracted from *Streptocarpus grandis* using the ONT DNA extraction method. DNA fragment size distribution assessed with Femto Pulse. **Figure S2.**  Results of *Streptocarpus grandis* DNA TapeStation analyses and library quality control (QC). DNA fragment size distribution assessed using TapeStation Genomic. a DNA integrity, b DNA fragment size distribution of DNA assessed with TapeStation Genomic. c Fragment size distribution of PacBio HiFi library. Two lanes on left are library duplicates.

**Additional file 2: Table S1.** Results of quality control (QC) of *Streptocarpus grandis* DNA extracted using the method applied for the *Streptocarpus rexii* ONT long-read sequencing method [15, 16]. **Table S2.** Results of quality control of the extracted DNAs. **Table S3.** Results of quality Quality control of (QC) DNA of two *Streptocarpus* species used for PacBio HiFi long-read sequencing. **Table S4.** Statistics of the PacBio HiFi long-read sequencing results.

## Data Availability

All data generated and analysed for supporting this protocol are included in the article.
