# Supplementary material for: A high quality, high molecular weight DNA extraction method for PacBio HiFi genome sequencing of recalcitrant plants
Source: Plant Methods. 2023 Apr 29;19:41. doi: 10.1186/s13007-023-01009-x (PMC10148486; doi:10.1186/s13007-023-01009-x)

Additional file 1:

Figure S1. DNA extracted from *Streptocarpus grandis* using the ONT DNA extraction method. DNA fragment size distribution assessed with Femto Pulse.

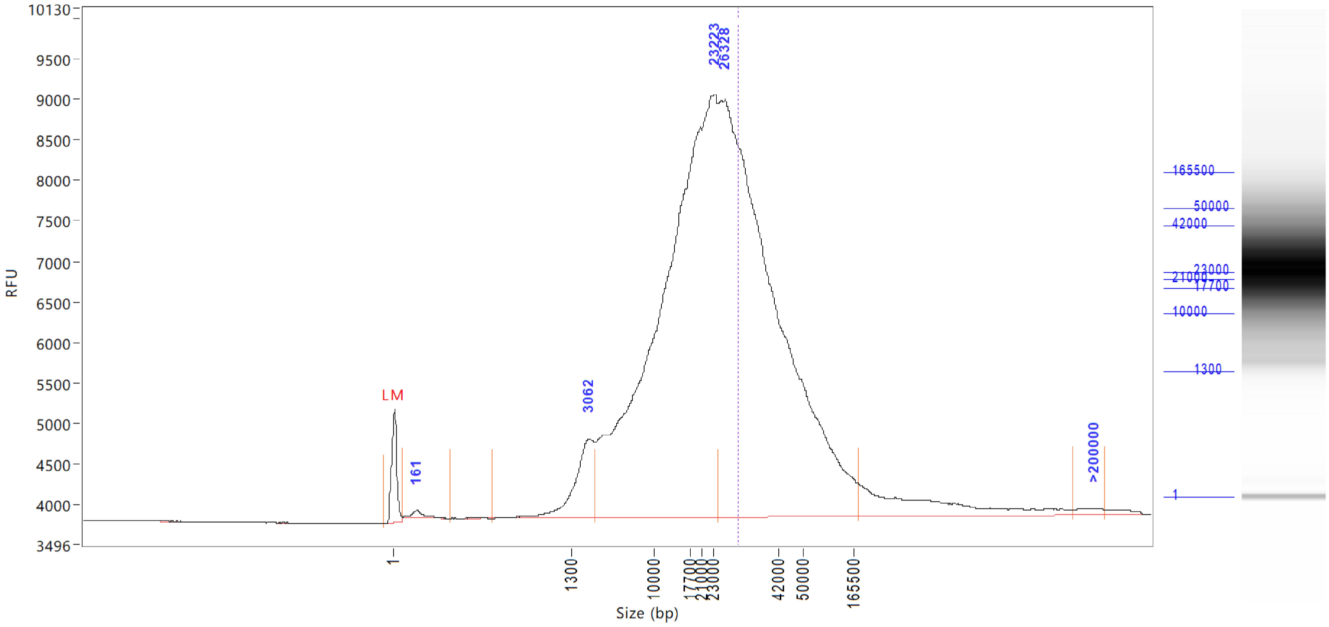

Figure S2. Results of *Streptocarpus grandis* DNA TapeStation analyses and library quality control (QC). DNA fragment size distribution assessed using TapeStation Genomic. **a** DNA integrity (A1 - ladder, B1 - DNA). **b** Fragment size distribution of DNA assessed with TapeStation Genomic. **c** Fragment size distribution of PacBio HiFi library. Two lanes on left are library duplicates.

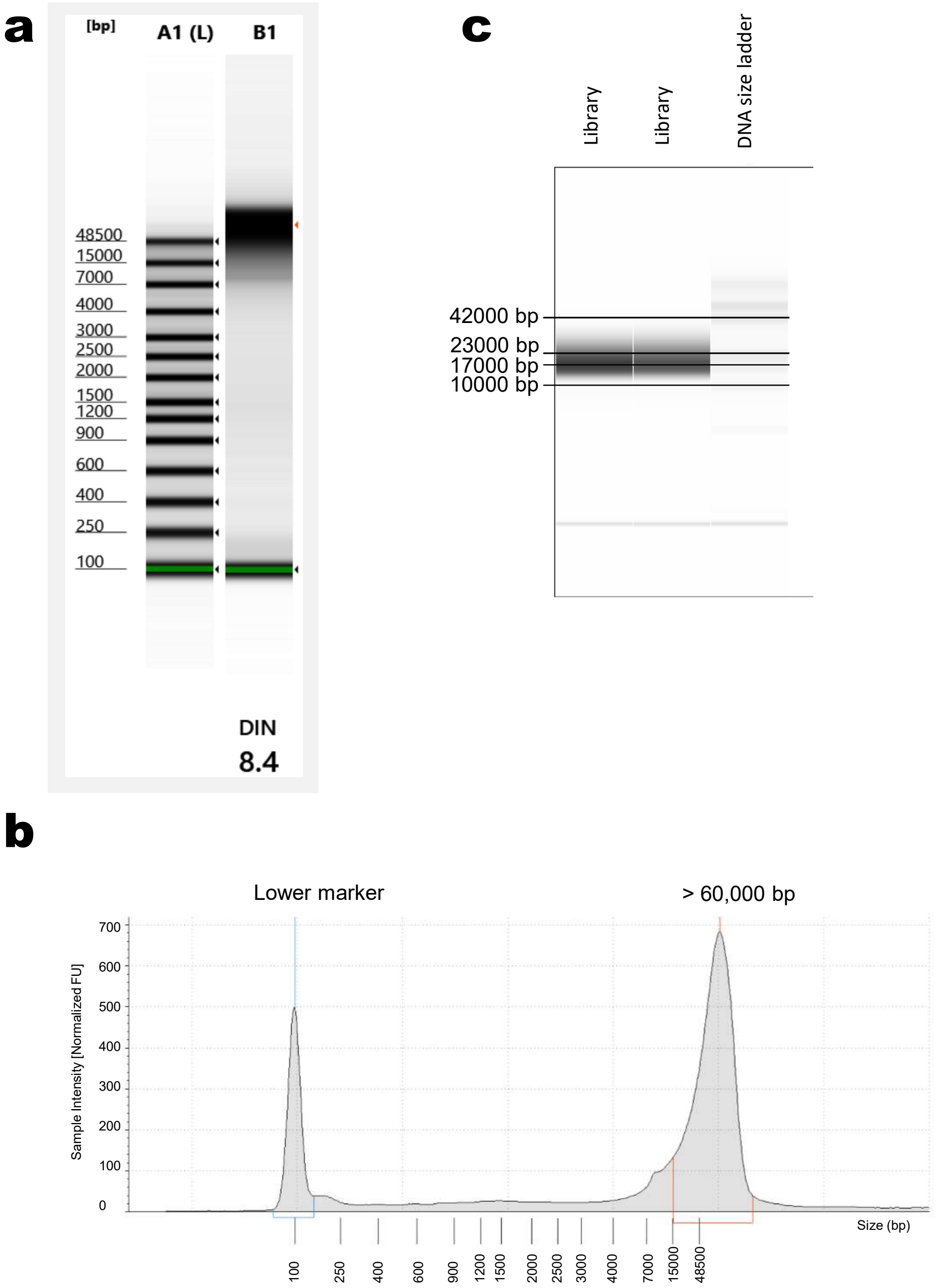

Supplement: Supplementary file 1 — Additional file 1: Figure S1. DNA extracted from Streptocarpus grandis using the ONT DNA extraction method. DNA fragment size distribution assessed with Femto Pulse. Figure S2. Results of Streptocarpus grandis DNA TapeStation analyses and library quality control (QC). DNA fragment size distribution assessed using TapeStation Genomic. a DNA integrity, b DNA fragment size distribution of DNA assessed with TapeStation Genomic. c Fragment size distribution of PacBio HiFi library. Two lanes on left are library duplicates. [file 13007_2023_1009_MOESM1_ESM.pdf]
