# Supplementary material for: A high quality, high molecular weight DNA extraction method for PacBio HiFi genome sequencing of recalcitrant plants
Source: Plant Methods. 2023 Apr 29;19:41. doi: 10.1186/s13007-023-01009-x (PMC10148486; doi:10.1186/s13007-023-01009-x)
Supplement: Supplementary file 2 — Additional file 2: Table S1. Results of quality control (QC) of Streptocarpus grandis DNA extracted using the method applied for the Streptocarpus rexii ONT long-read sequencing method [15, 16]. Table S2. Results of quality control of the extracted DNAs. Table S3. Results of quality Quality control of (QC) DNA of two Streptocarpus species used for PacBio HiFi long-read sequencing. Table S4. Statistics of the PacBio HiFi long-read sequencing results. [file 13007_2023_1009_MOESM2_ESM.pdf]

Additional file 2:

Table S1. Results of quality control (QC) of *Streptocarpus grandis* DNA extracted using the method applied for the *Streptocarpus rexii* ONT long-read sequencing [15,16].

QC device	Unit	Value
Qubit	Average concentration ng/µl	97.6
Nanodrop	concentration ng/µl	113.9
	A260/A280	1.74
	A260/A230	2.09
	Nanodrop conc. / Qubit conc.	1.17
TapeStation Genomic	DIN	8.8
Femto Pulse	%DNA > 15 kb	7.8
	%DNA > 25 kb	6.0
	%DNA > 50 kb	1.3

Table S2. Results of quality control (QC) of the extracted DNAs.
DNA QC results of the extracted DNA of two *Streptocarpus* species using the protocol established in this study. Two different lysis conditions were also compared. ON: overnight lysis, 4H: 4 hours lysis.

DNA ID	Lysis cond.	Taxon	Sample g	Qubit conc.	A260 / A280	A260 / A230	Nanodrop conc.	DIN	Vol µl	Total DNA ng	DNA_ng / Sample_g	GQN 15kb	GQN 25kb	GQN 30kb	GQN 40kb	GQN 50kb	GQN 60kb
KN327	ON	*S. grandis*	3.02	259.99	1.75	2.00	387.52	9.3	10	2599.9	860.89	8.9	7.8	7.2	4.9	2.0	1.7
KN328	ON	*S. grandis*	3.00	96.89	1.78	2.11	107.69	8.7	10	968.9	322.97	6.8	5.4	4.8	3.5	2.3	2.1
KN329	ON	*S. grandis*	3.16	60.50	1.76	1.75	67.40	7.5	10	605.0	191.46	5.6	3.8	3.1	1.8	0.9	0.8
KN330	4H	*S. grandis*	3.00	87.98	1.75	1.83	83.15	8.4	10	88.0	29.33	7.8	6.5	5.9	4.5	3.1	2.9
KN331	4H	*S. grandis*	3.12	219.62	1.76	2.03	207.69	9.2	10	2196.2	703.91	8.5	6.0	4.8	2.5	0.9	0.8
KN332	4H	*S. grandis*	3.06	75.37	1.80	1.90	67.91	9.4	10	753.7	246.31	9.1	8.2	7.7	6.1	3.0	2.5
KN333	ON	*S. kentaniensis*	3.01	11.38	1.87	1.67	12.89	7.6	10	113.8	37.81	6.4	4.7	4.0	2.7	1.7	1.6
KN334	ON	*S. kentaniensis*	3.03	35.40	1.82	2.00	34.08	8.2	10	354.0	116.83	6.8	4.7	3.9	2.4	1.3	1.2
KN335	ON	*S. kentaniensis*	3.10	14.84	NA*	NA*	18.19	7.9	10	148.4	47.87	5.9	4.4	3.8	2.7	1.7	1.6
KN336	4H	*S. kentaniensis*	3.00	21.80	NA*	NA*	27.09	7.4	10	218.0	72.67	7.2	6.2	5.8	4.9	3.7	3.5
KN337	4H	*S. kentaniensis*	3.07	47.00	1.86	1.94	43.82	8.7	10	470.0	153.09	8.1	7.1	6.6	5.2	3.7	3.4
KN338	4H	*S. kentaniensis*	3.08	42.67	1.81	2.04	46.61	9.5	10	426.7	138.54	8.7	7.8	7.4	6.3	4.4	4.0

***Concentration too low to evaluate the ratio accurately**

Table S3. Results of quality control (QC) of DNA of two *Streptocarpus* species used for PacBio HiFi long-read sequencing.

QC results of large DNA extractions using the method applied for PacBio HiFi long-read sequencing.

**QC criterion**	***S. grandis***	***S. kentaniensis***
Qubit conc. (ng/µl)	85.2	46.4
Nanodrop conc. (ng/µl)	120.0	53.6
A260/A280	1.92	1.72
A260/A230	2.38	1.91
DIN	8.4	8.5
Total volume (µl)	250	480
Total DNA (µg)	21.3	22.3
Starting material (g)	48	45
DNA (ng) / Sample (g)	443.8	495.6

Table S4. Statistics of the PacBio HiFi long-read sequencing results.
Sequencing results of PacBio HiFi long-read sequencing of two *Streptocarpus* species assessed with NanoPlot.

Taxon	*S. grandis*			*S. kentaniensis*		
	1st Cell	2nd Cell	Average	1st Cell	2nd Cell	Average
**Mean read length**	17,650.5	17,852.6	17,751.6	16,521.1	14,581.5	15,551.3
**Mean read quality**	33.1	32.5	32.8	32.9	33.7	33.3
**Median read length**	16,832.0	17,056.0	16,944.0	15,804.0	14,136.0	14,970.0
**Median read quality**	33.2	32.4	32.8	32.6	33.5	33.1
**Number of reads**	1,305,820.0	1,311,881.0	1,308,850.5	1,047,454.0	1,909,385.0	1,478419.5
**Read length N50**	17,774.0	18,026.0	17,900.0	16,839.0	14,615.0	14,227.0
**Longest read length**	57,931.0	52,048.0	54,989.5	46,286.0	44,537.0	45,411.5
**STDEV read length**	4,083.4	4,110.0	4,096.7	4,089.6	2,620.5	3,355.1
**Total bases**	23,048,385,353.0	23,420,426,059.0	23,234,405,706.0	17,305,132,054.0	27,841,761,735.0	22,573,446,894.5
